# Science communication on the public health risks of air pollution: a computational scoping review from 1958 to 2022

**DOI:** 10.1186/s13690-023-01031-4

**Published:** 2023-02-04

**Authors:** Elisabeth Pfleger, Christoph Adrian, Regina Lutz, Hans Drexler

**Affiliations:** 1 Institute and Outpatient Clinic of Occupational, Social, and Environmental Medicine, Friedrich-Alexander-Universität Erlangen-Nürnberg (FAU), Henkestrasse 9 – 11, 91054 Erlangen, Germany; 2 Department of Economics and Social Sciences, Chair of Communication Science, Friedrich-Alexander-Universität Erlangen-Nürnberg (FAU), Findelgasse 7/9, 90402 Nürnberg, Germany

**Keywords:** Air pollution, Public health, Science communication, Health communication, Information, Risk, Scoping review, Ultra-fine particles

## Abstract

**Background:**

Air pollutants are a health risk for the entire population. Particulate matter (PM) including the smallest fraction, ultra-fine particles (UFP), therefore continue to be the focus of scientific research in this area. To protect the population from the harmful effects of exposure to PM, communication and information of research results are of special relevance as individuals with heightened awareness of the harms of poor air quality are more likely to take action to improve their exposure.

**Methods:**

We conducted a scoping review of the scientific literature on science communication of public health information about risks associated with air pollutants to generate an initial over-view of existing research in this field. We searched the PubMed and Scopus databases and analyzed the data using a structured topic modeling (STM) approach.

**Results:**

The existing scientific literature dates back to 1958 but increases significantly from the 1990s onwards. Publications are mainly found in the discipline of environmental research and are primarily concerned with health effects. It is often stated that adequate communication of the results to the public would be important, but specific approaches are rare. Overall, the topic of risk communication seems to be underrepresented for both air pollutants and UFP.

**Conclusions:**

To protect public health, it is important to conduct more intensive science and risk communication related to scientific findings on the risks of air pollutants. For adequate communication and information, further research is needed to provide specific approaches that also involve the affected population and take different target groups into account. In addition, the effectiveness of communication efforts should also be analyzed.

**Supplementary Information:**

The online version contains supplementary material available at 10.1186/s13690-023-01031-4.

## Background

Clean air is essential for healthy living. The air we breathe is, however, contaminated with many pollutants which adversely affect human health [1]. As air pollution is now recognized as the single biggest environmental threat to human health, the World Health Organization (WHO) has recently published new global air-quality guidelines. These guidelines recommend maximum air quality levels for six pollutants, for which evidence on health effects from exposure is most advanced [2]. The most important air pollutants, also known as criteria air pollutants, with adverse effects on human health include particulate matter (PM_10_, PM_2.5_), ozone (O_3_), nitrogen dioxide (NO_2_), sulfur dioxide (SO_2_), carbon monoxide (CO), and lead (Pb) [3, 4]. Another air pollutant of increasing importance in scientific research is ultra-fine particles (UFP), the smallest components of PM [5].

The health effects of air pollutants are wide-ranging. Short-term exposure to air pollutants may promote respiratory effects [6], arterial hypertension or heart attacks [7, 8] and increased mortality has been observed from cardiovascular disease associated with air pollutants [9]. Furthermore, PM has been found to increase the risk of inflammatory diseases such as multiple sclerosis, and Tripathy et al. [10] recently found that chronic ambient exposure to PM_2.5_ contributes to this risk even in healthy adults [10, 11]. Long-term exposure to PM_2.5_ was found to further increase this risk [12]. Overall, long-term exposure to air pollutants over several years is associated with increased risk for cardiovascular and respiratory diseases [13–17]. Health risks associated with air pollutants can be observed at any age. As such, increased exposure to PM during pregnancy can contribute to preterm birth or higher newborn and early-childhood blood pressure [18–20]. Furthermore, the health effects caused by air pollutants can also become apparent at older ages. It has been found that late-life cognitive health may be affected by air pollution [21, 22]. Patten et al. [23] also recently found evidence in animal studies that chronic exposure to ambient traffic-related air pollution promotes Alzheimer’s dementia. This has also been observed in human studies [24, 25].

Based on WHO guidelines, various nations have therefore already derived statutory limits for many air pollutants which must be followed to avoid harmful effects on human health [26]. National air-quality guidelines also require states to monitor, document, and report on their air quality so that the public can be informed about air-pollution levels. In the European Union (EU), this is set out in the EU Directive 2008/50/EG, which, in most cities in the EU, is implemented through dedicated websites and reports [27]. Other commonly used tools to report air quality to the public are air-quality indices. These indices can be single- or multi-contaminant-oriented, which means they consider either only one pollutant or the combination of several pollutants to assess air quality. These indices allow to make the concentrations of pollutants and their harmful effects on health more understandable for the public. Thus, in case of poor air quality, protective measures against it can be taken by the public itself (e.g., reduce stay outdoors or close windows). The method on which the index is based and how it is calculated varies from country to country, which can make it difficult to compare air quality between nations [28, 29]. Although limits and mitigation measures are already in place, air pollution in Europe continues to reduce average life expectancy by about 2.2 years [30]. Globally, an estimated 4.2 million people die due to ambient air pollution each year [31]. Reasons for this may include not only anthropogenic sources but also the interaction of various processes in the atmosphere. Meteorological influences, for example should be mentioned in this context, since some weather conditions can lead to the accumulation of pollutants [32]. Natural sources also contribute to air quality, such as desert dust, pollen, volcanic eruptions, sea salt aerosols, forest and bush fires, or biogenic volatile organic compounds emitted by plants [33].

Overall, air pollution in Europe has declined in recent decades, while traffic and industrial production have increased. Improvements in fuel quality and new technologies for exhaust-gas cleaning have made a significant contribution to this development [34, 35]. The SARS CoV-2 pandemic and the associated reduced emissions from road or air traffic, for example, also led to a short-term reduction in pollutants in many places [36]. However, a long-term and permanent improvement in air quality can only be achieved with targeted clean-air policies, such as the implementation of measures from clean-air plans which focus on permanent changes [37]. Moreover, air pollution does not only take place outdoors. People can also be exposed to many air pollutants indoors. Here, however, there is the possibility that individuals can influence their exposure by, for example, opening windows or stopping combustion processes, such as cooking using open fires or smoking [38]. In instances where individuals can control their environment, public understanding of the risks of air pollution may have a greater impact on improving exposure because those who are more aware of the risks are often more likely to take action to improve their exposure [39, 40].

Still today air pollutants continue to represent an important field of research, especially since not all fractions have been sufficiently researched to date. The reported health effects mostly involve PM_10_ or PM_2.5_ [7–10, 12–15, 18–23]. Due to their small size of less than 100 nm, UFP have negligible mass compared to PM_10_ and PM_2.5_, but contribute significantly to the total number of particles in the atmosphere [41]. In contrast to PM_10_ and PM_2.5_, data for UFP are still insufficient with respect to human health effects. Most evidence comes from animal studies or short-term human-exposure studies [42]. Therefore, a still-unresolved question in air-pollution research is to what extent UFP contribute to the aforementioned risks. This research gap is currently being addressed by the research network BayUFP, which is funded by the Bavarian State Ministry for the Environment and Consumer Protection [43]. In this context, it is not only important to obtain scientific findings, on health effects for example, but also to make these available in a suitable format and to communicate them adequately. In research, this is primarily achieved by means of scientific publications and lectures, which are usually addressed to a scientific community [44]. Since air pollution, including the field of UFP, is an issue that affects the entire population, it also requires appropriate communication of research results and thus, most importantly, the health risks to the public. Education and information on environmental-health risks is part of the field of environmental medicine [45]. In this regard, educating the public about environmental-health risks is often more difficult than educating individual patients about specific medical interventions. The reason for this is that public education usually does not take place in individual, direct conversations, but at the societal level, and is therefore more fraught with uncertainty and subject to misinformation [46]. To conduct adequate science and risk communication, it is considered essential to first review the existing literature. Since the literature on UFP with regard to communication is very scarce, and since UFP represent only one part of air pollution in total, an analysis of the literature on air pollutants as a whole will be used as a basis. Due to the heterogeneity of the literature, a precise systematic review of the literature with a specific research question is not considered to be appropriate at this point. In order to obtain an initial overview and to be able to conceptually limit topic areas to the field of communication on air pollutants, the analysis will initially be carried out within the framework of a scoping review with a computer based Structured Topic Modeling (STM). This scoping review aimed to identify and categorize the published scientific literature on science communication of public health information in the context of air pollution.

For mapping a research field, it is first necessary to describe the stock and development of previous publications [47]. Thus, in a first step we illustrated the evolution of science communication and information in the context of air-pollution research across time and publication types. In addition, we analyzed the disciplines from which the publications originate to map the most relevant research areas in which communication and information on air pollutants has already been conducted. We established the following research question: To what extent has science and risk communication / information / public perception related to air pollutants been published (i) over time, (ii) across different publication types, and (iii) across different disciplines?

In a second step, we attempted to systematically identify the core topics that receive attention in interdisciplinary research using a Topic Modelling (TPM) approach. This can help to pool the knowledge generated in different disciplines on these topics and to prepare and facilitate a more detailed systematic literature review into the identified topics. Examining the evolution of these topics over time can tell us whether and how certain topics dominate the field and whether others have lost or gained prominence. Our second research question is therefore: What are (i) the most important topics that have been studied in this context and (ii) how are these topics distributed over time?

The purpose of this scoping review is to summarize existing research, identify research gaps and present recommendations for future research regarding science and risk communication related to air pollution specifically as relates to UFP.

## Methods

Communicating environmental risks from air pollution is a very broad issue with a number of different approaches.

As a systematic review approach with manual search and coding would be very resource-intensive and outdated upon completion, we applied computational methods. Consequently, a much larger amount of data can be included with this approach, and the literature can be described more comprehensively to obtain an initial overview for more detailed research [48].

### Analytical approach

Topic Modelling is a method from the field of Natural Language Processing (NLP) for the exploration of larger text collections. The automated, unsupervised process attempts to uncover latent thematic structures (topics) within a large number of documents (corpora) by computing statistical models based on clusters of co-occurring words [49]. Topics are defined as a multinomial distribution of words, where each word has a probability (ß) of being assigned to a particular theme. A document represents a distribution of all topics with different (γ-) probabilities [50]. The application of TPM to journal-article abstracts is consistent with previous research [51–54] and is based on four criteria [55]: (i) abstracts represent a concise summary of the article in terms of research aims, problems, and major findings [56, 57], minimizing the likelihood of identifying marginal or random topics, and therefore (ii) allow for a decent comparison in terms of format and style guidelines across journals in contrast to full texts. In addition, (iii) abstracts are freely available and (at least in both databases used here) (iv) can be collected automatically (via Application Programming Interface (API)). This paper used the STM framework [58] for topic identification, which, unlike other common algorithms such as Latent Dirichlet Allocation (LDA) [49], allows for the consideration of metadata when estimating topics. We included the year of publication as metadata. The use of spectral initialization is intended to prevent the usual variation of topic models (e.g. depending on the seed) and to keep the results robust.

### Data collection and processing

This scoping review was conducted following the Preferred Reporting Items for Systematic Reviews and Meta-Analyses Extension for Scoping Reviews (PRISMA-ScR) Statement [59]. The search terms were systematically developed in an iterative process that consisted of several manual validations and adjustments. The final string includes both a specific focus on risk communication and PM as well as more general, overarching terms like public opinion or health communication (Table 1, Additional file). To cover a broad spectrum of research, the string was applied to two pertinent databases: Scopus and PubMed. The corresponding API, a set of commands, functions, protocols and objects that facilitates the communication of different applications with each other and in this case simplifies the access to the data, of each database was used on April 30, 2022 to search articles’ titles, abstracts, and keywords, without any additional time restrictions; abstracts and available metadata for all results were downloaded as an .xml file (for additional information see Table 2, Additional file). The API was accessed using two different R functions: *pubmedXML* (https://github.com/christopherBelter/pubmedXML) and *scopusAPI* (https://github.com/christopherBelter/scopusAPI). To ensure comparability and allow proper functionality of the automated content analysis, only publications in English were included. Publication formats included journal articles, reviews, and conference proceedings. Conference papers were included as we hoped to find approaches that were initially developed and presented e.g., at conferences, but not (yet) finalized and published but could also play an important role in the further development of risk communication and information. Our decision was based on the suggestions from Sherer and Saldana [60]. After downloading the data, all duplicates (based on the title), entries which lacked an abstract, as well as all papers not yet published were excluded. All analyses were based on the articles’ abstracts and metadata (e.g., title, keywords, year) (for additional information see Table 2, Additional file). Prior to analysis, additional pre-processing of the data is necessary. As (very) short documents negatively influence topic modelling performance [61], we removed *n* = 28 abstracts with less than 50 words. Additionally, to ensure comparability, all abstracts (*n* = 14) with more than 1000 words were excluded. To provide context and emphasize the noteworthy content of the articles, the corresponding title and keywords were added to the abstract. The corpus was built with the *quanteda* package [62], taking into account various commonly used pre-processing steps (removal of punctuation, numbers, symbols and stop words). In addition, words were lemmatized using the *spacyr package* [63], 2- and 3-g were added to the corpus, and, based on the guidelines of Maier et al. [64], all words and N-grams (contiguous sequence of n words) that occur in more than 99.9% or less than 1% of the documents were removed.

### Model selection

**Fig. 1:**
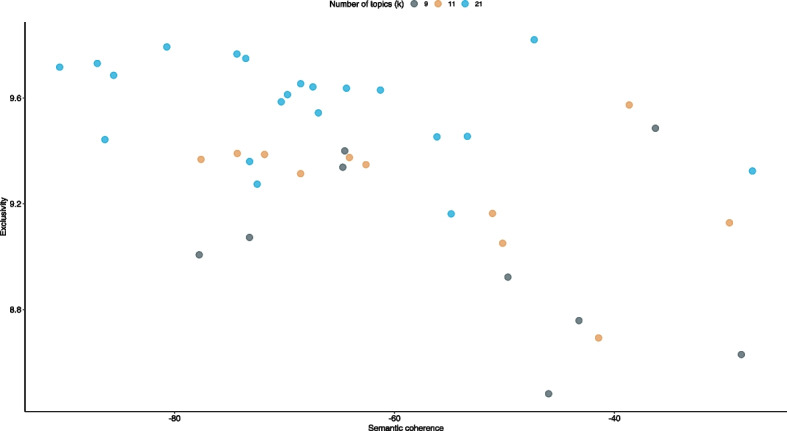
Comparison of exclusivity and semantic coherence for different topic numbers (k = 9, k = 11 and k = 21)

A key challenge when using TPM is determining the optimal number of topics [k]. In general, as the number of topics increases, so does their specificity. The challenge is to find a balance between exclusivity and coherence. On one hand, the extracted topics should not combine different aspects that should actually be considered independently. On the other hand, it would be difficult to make a meaningful distinction if too many topics were included [64]. The *stm package* (or specifically the *stm function*) provides an option to have the number of topics estimated automatically using an algorithm from Mimno and Lee [65]. However, this estimate should be understood as a starting point for further analysis rather than a statistically guaranteed optimum number of topics [58]. This paper elicited the adequate number of topics using an iterative process, a combination of automatic and “substantive” search [66]. In the first step, 100 models with randomized seeds were estimated using the aforementioned automatic topic-selection strategy (Mimno-Lee algorithm) to check the consistency of the “optimal” number of topics. The results were used to derive the maximum number of topics for the “manual” search performed in the second step, which first estimated a set of models (10 > k > 90 with constant seed in steps of k = 1) and then evaluated them using four different statistics: held-out likelihood, lower bound, residuals, and semantic coheres (for additional information, see Fig. 1, Additional file). The main criterion for selecting the final number of topics is semantic coherence [67], which maximizes when the most prominent words of a topic occur frequently together [58] and correlates with human (expert) judgments of topic quality [67]. Semantic coherence, however, only refers to the internal consistency of a topic and neglects whether topics are similar; it easily arises when a few topics are dominated by very common words. As such, the final assessment of topic quality should additionally consider the exclusivity of topic words [68]. In the first step, the models with 9, 11, and 21 topics were selected because of their relatively high semantic coherence, while maintaining a held-out likelihood as high as possible and residuals and lower bounds as low as possible [41]. Based on the comparison of semantic coherence and exclusivity (see Fig. 1) combined with additional information (for example, its top terms and top articles representing the topic), the eleven-topic model was selected for the final analysis including the following topics: “risk communication”, “air quality management”, “climate change and adaptations”, “energy”, “prediction models”, “epidemiological analyses”, “respiratory diseases”, “pregnancy and birth”, “pollutant sources and concentrations”, “filters and membranes” and “preclinical analyses”.

## Results

### Study selection

The initial search of the databases returned 11052 hits. Respective results for each database are listed in Table 1.

**Table 1:** Searched databases in the period 1958 – 2022 and hits

Database	n	%	% cum
PubMed	3941	35.66	35.66
Scopus	7111	64.34	100

**Fig. 2:**
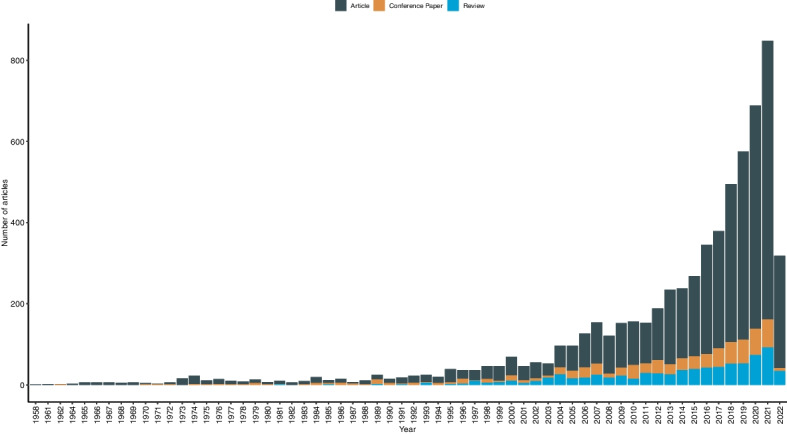
Publications over time (1958 – 2022) by document type (articles, conference papers and reviews) with *n* = 6423 references in total. *Note*: the decrease in all document types in 2022 is most likely due to data collection in May 2022

Due to the a priori defined inclusion and exclusion criteria, and after removing duplicates a final sample of *n* = 6467 potentially relevant references was obtained. These results were included in the further analysis. A flow diagram of this process can be found in Fig. 2, Additional file.

### Publication behavior in the field: over time, across publication types, and cross disciplines

After technical cleaning and filtering, our search provided a total of 6423 references covering a publication period from 1958 to 2022 (see Fig. 2).

At the beginning of this period, however, only very few publications were available. Starting around the mid-1990s, an increase in availability can be observed which continues steadily and reaches a peak in 2021.

We limited our search to original articles, conference papers, and reviews. Regarding the development of these three scientific document types over time, it is evident that original articles clearly dominate and are continuously increasing, as are review papers which refer to them. Conference papers, on the other hand, are proportionally much less available over the entire period, but also with a continuously increasing trend.

For analyzing the disciplines in which the identified publications occurred, we examined the journals in which the references were published. The final sample consists of 6423 references from 2041 journals, with 108 journals covering almost 50% of all articles. For the analysis of the disciplines of the publications, we focused on the top ten journals, as they contain almost one fifth of all publications, and examined their scope and objectives (see Table 2).

**Table 2:** Top 10 journals with shares within the sample of *n* = 6423 references in the period 1958 - 2022

Journal	n	%	% cum
International Journal of Environmental Research and Public Health	204	3	3
Science of the Total Environment	202	3	6
Atmospheric Environment	174	3	9
Environmental Research	136	2	11
Environment International	105	2	13
Environmental Health Perspectives	100	2	14
Environmental Science and Pollution Research	100	2	16
Environmental Pollution	89	1	17
Journal of the Air and Waste Management Association	86	1	19
Environmental Science and Technology	84	1	20

Regarding the disciplines of the publications, it is interesting to note that the most represented journals are primarily related to environmental sciences, while publications specifically related to health or risk communication and information were not found among the top ten journals in this sample. Moreover, there were also numerous publications in the field of atmospheric research.

### Most important topics

In this section, we present the topics identified by STM, as well as representative related studies.

We selected a model with eleven topics into which all the identified literature was classified. For each topic, we first selected the five most representative abstracts, based on the highest γ-probability and examined their titles, abstracts, and keywords as well as the top 10 lemma, the base form of a word, that is used as the dictionary entry, to which inflected forms (such as plurals, verb tenses, etc.) are related, (see Table 3) to find a suitable topic name after multiple rounds of discussion.

**Table 3:** Topics and topic groups identified through the structured topic modelling with topic number k = 11 within the literature published in the period 1958 - 2022

#	Topic name	Topic Group	Prevalence	Top 10 keywords (lemmatized)
3	Risk communication	Risk communication	0.1466	pollution, air, air pollution, health, risk, public, environmental, public health, study, assessment
8	Air quality management	Air quality management	0.1053	air, quality, air quality, indoor, health, monitoring, public, indoor air, standard, pollutant
5	Climate change and adaptations	Environmental protection, climate, sustainability	0.1000	health, change, climate, public, urban, public health, impact, covid-19, climate change, environment
10	Energy	Environmental protection, climate, sustainability	0.0894	emission, health, benefit, energy, impact, reduction, reduce, air, cost, control
7	Prediction models	Prediction models	0.0985	model, air, datum, concentration, spatial, study, air quality, quality, base, result
9	Epidemiological analyses	Health impacts	0.0934	mortality, air, pollution, air pollution, increase, disease, pollutant, association, study, term
6	Respiratory diseases	Health impacts	0.0820	child, asthma, disease, respiratory, factor, health, study, lung, risk, cancer
4	Pregnancy and birth	Health impacts	0.0609	exposure, study, association, health, smoke, outcome, associate, birth, population, risk
11	Pollutant sources and concentrations	Substance analysis and filters	0.0781	emission, source, concentration, traffic, pollutant, vehicle, carbon, study, urban, level
1	Filters and membranes	Substance analysis and filters	0.0739	particle, particulate, air, airborne, aerosol, sample, filter, matter, particulate matter, study
2	Preclinical analyses	Preclinical analyses	0.0719	pm2.5, particulate, matter, particulate matter, exposure, concentration, fine, m3, μg, health

Note: Prevalence describes the average probability of assigning a certain topic to a certain document

In addition, due to the similarities and commonalities of certain topics, topic groups were formed. The combined topics were no longer individually considered, resulting in seven topics for further analysis. We then reexamined the titles, abstracts, and keywords with respect to our research question. Topics not containing at least one article among the top 5 references related to the research questions were excluded from further analysis. A list of all initially identified topics with the corresponding top 5 articles can be found in Table 3, Additional file. The following topics were included in the further analysis: *health impacts; environmental protection, climate, sustainability; risk communication; prediction models; air quality management* (see Table 4).

**Table 4:** Topic groups of the sample identified through structured topic modelling with topic number k = 11 and merged manually

Topic (group)		All topics		Only included topics	
	n	%	% _**cum.**_	% _**valid**_	% _**valid cum.**_
Health impacts	1529	23.81	23.8	30.2	30.2
Environmental protection, climate, sustainability	1273	19.82	43.6	25.1	55.3
Risk communication	947	14.74	58.4	18.7	74.0
Prediction models	664	10.34	68.7	13.1	87.1
Air quality management	653	10.17	78.9	12.9	100.0
Substance analysis and filters	998	15.54	94.4		
Preclinical analyses	359	5.59	100.0		

Note: n = Number of references, with the respective topic as the main topic (based on the highest γ-probability) / % = proportion of the articles in the total sample (n = 6423) / % _valid_ = proportion of the articles included in the final analysis (*n* = 5066)

### Health impacts

The largest topic is *health impacts*, consisting of *n* = 1529 references (30.2% valid) It merges the initially identified topics *pregnancy and birth*, *respiratory diseases,* and *epidemiological analyses*. This topic covers human diseases related to exposure to air pollutants. Risk factors and effects are analyzed which exist or can occur in utero and postnatally [69–72], diseases in childhood [73], as well as diseases that can occur throughout the entire lifespan, such as respiratory diseases [74, 75], including chronic obstructive pulmonary disease (COPD) [76] and tuberculosis [77] or cardiovascular diseases [75, 78]. The majority of these studies focus on the medical causes and effects of exposure to air pollutants. Some analyses further conclude that the results obtained are important for public health or highlight that they are a public health concern [70–72, 75, 76, 78]. In this regard, Santri et al. [69] noted that it is particularly important for of the affected population to understand the identified impacts. Guo et al. [77] highlighted the need for public-health action in studying exposure to household air pollution, noting that reducing the incidence of tuberculosis for high-altitude residents should be a public-health priority. Rinne et al. [74] also concluded the need for public-health action in order to reduce respiratory illnesses from exposure to biomass smoke in the home. Only Boothe et al. [73] mentioned a specific opportunity for communication or information, concluding in a review on the association between exposure to near-housing traffic and cancer in children that public-health messages would be important to reduce traffic exposures in the population in general.

### Environmental protection, climate, sustainability

The topic *environmental protection, climate, sustainability* comprises *n* = 1273 articles (25.1% valid). It compiled of the two initially identified topics *climate change and adaptation* and *energy*. A key aspect of this merged topic is climate change, in which air pollution also plays a substantial role. In this context, adaptation strategies and mitigation measures necessary to counteract the negative effects of climate change are examined. These also require adequate public (health) communication or information to raise awareness of this issue [79–81]. In this context, Poutiainen et al. [79] identified some deficits, such as little evidence of substantial interventions or too little consideration of vulnerable groups. Various actions are mentioned to inform and educate the population through communication regarding climate change and its negative impacts: civil society organizations [79], nurses [82], and political institutions [81], such as the Ministry of Health [83]. To this end, Wu and Lee [82] have proposed an approach to improve communication of risks to the public by integrating appropriate components into the curriculum and training of nurses, who are on the frontline of medical care and thus of health communication. However, there are also specific tools that can be used by the population itself to obtain information about air quality [80]. Su [80] proposed a Google-based online intervention tool as one way of supplying information. This tool suggests outdoor activities to vulnerable groups taking into account the prevailing air quality [80]. Overall, it was found that climate goals and environmental objectives can only be achieved if the public is also made aware of the associated risks [81].

### Risk communication

In our sample, *risk communication* emerged as the third largest topic with *n* = 947 references (18.7% valid.). Different approaches to communicating risks associated with air pollution have been assessed. Some studies included specific measures that involved the concerned population in the research on communication [84, 85]. As such, the analysis of communication was performed using different approaches: While Huang and Yang [85] investigated how a form of communication specifically developed for risk communication affects the population, Börner et al. [84] analyzed influencing factors on risk perception of the public. Both studies aimed to either develop more appropriate communication strategies based on their scientific findings or to analyze their effectiveness and modify them accordingly [84, 85]. Börner et al. [84] also examined factors related to exposure to heightened risk perceptions and identified information-seeking behavior, political support, and individual mitigation measures. Further analyses focused on different populations [84–87]. Künzli and Perez [86], Huang and Yang [85] and Fischer et al [87]. included the general population into their research, whereas Börner et al. [84] selected adolescents as a special target group. Various types of media have been used to investigate the potential impact of communication and information, such as cinematography [85], photography [84], and print media (e.g. newspapers) [88]. Within these media, Lui and Zhang [88] found cultural differences in newspaper reports about health risk and information. In addition, using air pollution as a pedagogical example, Künzli and Perez [86] examined the discipline of “evidence-based public health,” which aims to act as a link between scientific research and public health to inform the public about scientific findings. Furthermore, Fischer et al. [87] described the development of approaches for the quantitative examination of air, which aims to generate data based on which informative statements can be made available, facilitating public health risk assessment.

### Prediction models

The topic *prediction models* includes *n* = 664 references (13.1% valid). Air-quality prediction and monitoring models represent the core of this topic. Here, information on air pollutants and pollution is made available in processed form through various mathematical calculations. A common feature of these models is machine learning or artificial intelligence, on which the prediction calculations conducted are based [89–92]. The prediction of air quality and pollution within these models thereby includes different pollutants, either analyzing only a single pollutant, like O_3_ [89] or NO_2_ [91] or different pollutants in parallel, for example PM_10_, SO_2_, CO, NO_2_ and O_3_ [90, 92]. In this context, Wu and Lin [92] optimized a model that includes the air-quality index (AQI), a summary value based on different air pollutants that evaluates air quality and can provide accurate information reflected in a single value. The models differ slightly based on their intended use. Some models are used to monitor current air quality [89], while others are used to forecast air pollution over time [90, 91]. In addition, Yan et al. [90] also mentioned warning the public as an intended goal of these systems.

### Air quality management

*Air quality management* represents the smallest topic in our sample with *n* = 653 references (12.9% valid). Air quality was examined in different settings: indoor air quality [93–95] as well as ambient air quality [96, 97]. Analyses include management of specific single pollutants like formaldehyde [94] and the management of mixtures of selected environmental air pollutants [96, 97], as well as general air quality without specific reference to any individual pollutants [93, 95]. The topic focuses primarily on the examination of public laws and policies [93, 96, 97]. They are partially addressed to specific target groups which must derive actions based on these regulations [93, 96]. For example, the research of Chan [93] addressed planners, contractors, developers, building owners and management companies, while Longhurst et al. [96] focused on local authorities. Various countries and their different regulations were considered, including Portugal [94], Singapore [93], the United Kingdom [96], and Texas in the United States [97]. Furthermore, Evagelopoulos et al. [95] developed special applications to assess air quality which determine the prevailing indoor air quality and present the obtained results with visual effects. This paper aimed to make data about air quality easier to understand for the general population [95].

### Changes over time

**Fig. 3:**
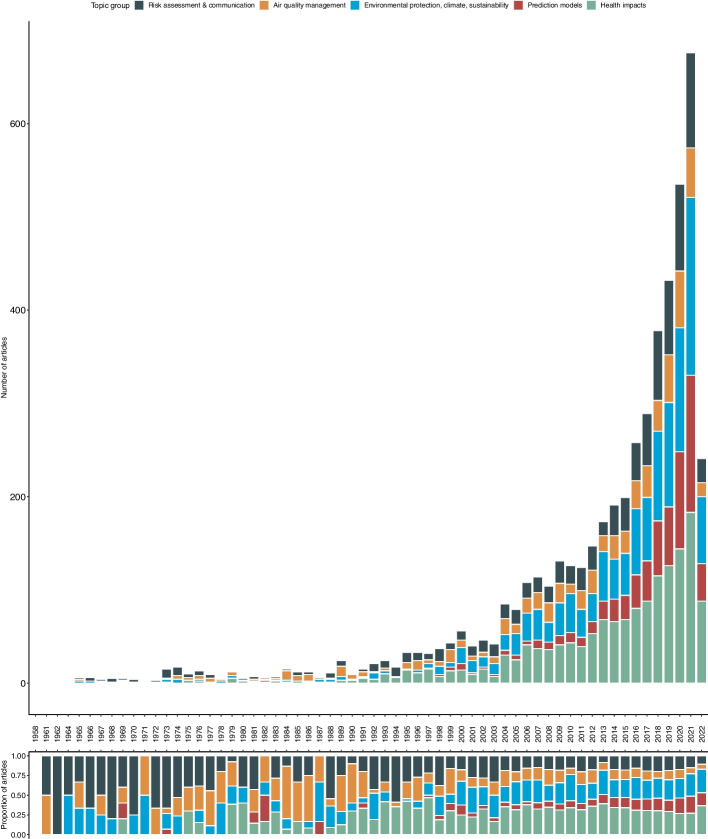
Distribution of valid topics identified through the structured topic modelling and merged manually over time by number of articles and proportion of articles (1958–2022) with *n* = 5006 references. *Note:* The decrease in all topics in 2022 is most likely due to data collection in May 2022

Looking at the distribution of the valid topics over time, we see that all topics appear very early in the timeline and have increased by number of articles over the entire period up to 2021 with fluctuations throughout (see Fig. 3). Since the 1990s, there has been a noticeable increase in the number of publications especially in the topics *health impacts* and *risk communication*. Overall, the topics *health impacts* and *environmental protection, climate, sustainability* are the dominant topics over time by this metric.

In terms of the proportion of articles in the sample (see Fig. 3), the topic of *health impacts* accounts for the largest proportion, particularly from the 2000s onwards, with some fluctuations. In contrast to the other topics, the topics of *risk communication* and *prediction models* exhibit a decrease in their shares over the entire period. While their shares still dominated with their shares up to the 1990s, they decrease continuously from then until 2021.

### UFP in the literature

**Fig. 4:**
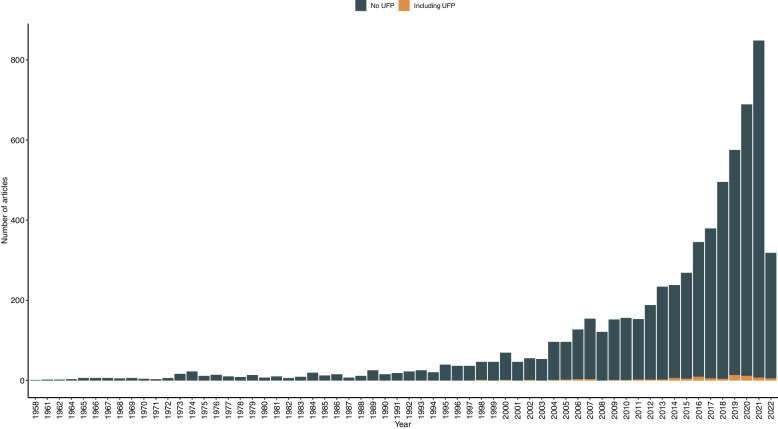
Numbers of publications over time with (*n* = 84) and without (*n* = 6339) including the term “UFP” or “ultra-fine particles” (1958 – 2022). *Note:* The decrease in 2022 is most likely due to data collection in May 2022

As UFP are currently an important subject of air-pollution research and the risks have not yet been conclusively researched and will thus also become part of risk communication in the future, we additionally analyzed our data on references especially concerning this fraction of pollutants. Out of the total 6423 references identified by the database search, only 84 references include the term “UFP” or “ultra-fine particles” in their title, abstract, or keywords, which accounts for an approximate 1% of the total sample. Regarding the development of publications over time, the first publication which explicitly mentions UFP was published in 1998. From then on, there was only a very small increase in the number of publications up to 2021 (see Fig. 4).

Publications which include the terms “UFP” or “ultra-fine particles” originate primarily from the discipline of environmental research (see Table 5) and are largely with the disciplines and journals in the general analysis of disciplines indicated in Table 2 consistent.

**Table 5:** Top 10 journals with shares within the sample of n = 6423 references containing the terms “UFP” or “ultra-fine particles” within the literature identified in the period 1958 – 2022

Journal	n	%	% cum
Atmospheric Environment	9	11	11
Science of the Total Environment	5	6	17
Environment International	3	4	20
Environmental Pollution	3	4	24
International Journal of Environmental Research and Public Health	3	4	27
Journal of the Air and Waste Management Association	3	4	31
Aerosol and Air Quality Research	2	2	33
Building and environment	2	2	36
Environmental Research	2	2	38
Environmental Science and Technology	2	2	40

Regarding our topic model, references containing UFP were mostly found in the topics *filters and membranes*, *pollution sources and concentrations,* and *preclinical analyses,* but did not appear among the manually checked top 5 references in any of the identified topics (see Table 6).

**Table 6:** Topics of the sample including the terms “UFP” or “ultra-fine particles” identified through structured topic modelling with topic number k = 11

Topic	n	%	% cum.
Filters and membranes	24	28.57	28.57
Pollutant sources and concentrations	21	25.00	53.57
Preclinical analyses	9	10.71	64.29
Risk communication	8	9.52	73.81
Respiratory diseases	6	7.14	80.95
Air quality management	4	4.76	85.71
Epidemiological analyses	3	3.57	89.29
Energy	3	3.57	92.86
Prediction models	2	2.38	95.24
Pregnancy and birth	2	2.38	97.62
Climate change and adaptions	2	2.38	100.00

In an additional step, we manually analyzed the titles, abstracts, and keywords of the identified 84 references to UFP on approaches containing communication or information. We identified eight references which address our research question [98–105]. Studies focusing on UFP mainly point out that they pose a potentially serious public health threat that should be communicated to the concerned population [99, 101, 103]. Various actions were cited as responsible for this task, including environmental-health agencies [99], governmental actors [101, 102], and health educators [103]. Two studies included specific target groups in their research, such as children exposed to UFP indoors and outdoors [103] or law-enforcement personnel exposed to traffic pollutants [102], and pointed out the importance of risk communication but neglected to suggest how this could be adequately implemented. In addition, predictive models for UFP were also presented in the literature [100, 104], for example by Jingshi Li et al. [100] which use satellite-based techniques for the determination of particulate-matter concentration and the associated risk to public health. Only Wong et al. [105] and Brugge et al. [98] developed specific approaches for science communication about public health information, by including the affected population in their research. Wong et al. [105] proposed an approach to visualizing air pollution by using maps of traffic pollution. This visual approach was intended to promote environmental health literacy about highway pollution and was achieved by educating adults on the use of these maps [105]. Brugge et al. [98] used fact sheets to communicate the risks of traffic-related air pollution to an adult population. Thematic analysis of transcripts was used to explore how these fact sheets could be improved. Improvements included increasing text size, adding more graphics, restructuring the text, and providing more technical details; this approach also considered the health literacy of the target population [98].

## Discussion

### Discussion of the results

The scoping review includes 6423 references which address science communication of public health information about risks associated with air pollution. By using a STM-Framework, 5066 references could be mapped into 5 final themes. When analyzing the development of the identified literature over time, it became apparent that although research on this topic has already been conducted since 1958, it was only available to a very limited extent during that time. A significant increase in publications across every publication type can be found from the 1990s onwards. This development is most likely due to effects of the change in the scientific system. The digitization that began with the invention of the internet led to a change in the availability of databases, resulting in a higher frequency of publications [106]. Nevertheless, research on communication and information related to air pollution seems to remain of increasing relevance due to the continuous increase in the number of publications to date. Regarding the disciplines of the publications, it is interesting to note that the most represented journals were primarily associated with environmental sciences, while publications focusing specifically on health communication, risk communication and information were not found among the ten journals with the largest shares in this sample. There are many possible reasons for this finding. Many publications simply state, in an introductory sentence, that air pollutants are a public health problem without further exploring this problem in their work, such as appropriate communication approaches and/or strategies [107–110, 112, 112, 112, 112]. These publications were initially identified due to our search string but were excluded from further analysis because they did not contribute to our research question based on the screening of their titles, abstracts, and keywords.

In total only a few studies in our sample focused on specific communication approaches to inform the public about risks associated with air pollutants. Some work aimed at developing appropriate communication strategies or analyzing their effectiveness [85, 86]. Few studies included the affected general population, e.g., the Chinese population in their investigation [85, 86] or considered different target groups, for instance adolescents or people in public health institutions [84, 87]. Since air pollutants are a risk that affects the entire population, it is necessary, on one hand, to include the population as a whole more intensively into risk communication research. It should be noted, however, that the population not only differs significantly in terms of sociodemographic characteristics such as age, gender, income, education level, and migration background, but also by values and life goals. These differences result in different communication needs, such that different target-group-specific approaches should be taken into account as well. This can be done in a variety of ways, e.g., by dividing the population by social strata when planning suitable communication strategies [112].

Involving experts familiar with the topic into the process of communicating risks to the affected population is also an appropriate approach that has been explored in the literature. This can be done in two ways: top-down by involving political actions [81, 83] or bottom-up by involving professionals who have proximity to the affected population, such as nurses [82]. These two approaches seem promising, since both environmental-protection authorities and medical professionals enjoy a high level of trust among the population when it comes to obtaining information about air pollution [112, 112].

The general population itself is increasingly prominent in communication process [112]. At the same time, many studies have focused primarily on generating data for quantitative investigation of air quality, which can then be used as a basis for deriving general information on air quality [87], predictions [89–92], or guidelines and legal texts [93, 96, 97]. However, this is only a first step to facilitate the estimation of health burdens or to provide information for the general population or even selected target groups. The classical bilateral sender-receiver model [112] needs to be considered more dynamically, as exchanges are fundamentally and strongly based on two-way or multipath communication [112]. Active information or communication to the population or an analysis of effectiveness in terms of understanding or perception were not investigated by most publications. Presenting data in a simple and accessible way (e.g., through visualizations with the help of modern communication methods and devices like smartphone apps) is therefore a good way to increase comprehension, especially for non-experts to enable them to take action to protect their health [112]. Nonetheless, by providing only data to facilitate the assessment of air pollution, it remains unclear whether these data are directed at laypersons or experts and whether they are understood by the affected target group.

Interestingly, given our research question, risk communication did not prove to be the dominant theme – neither over time nor overall. Most publications in our sample were linked to the topic *health impacts*, followed by *climate, environmental protection, sustainability*. Nevertheless, approaches concerning communication or information of risks in the context of air pollution were mostly represented by the topic *risk communication*. Even though the topic *health impacts* represented the largest proportion of our sample, no specific focus on communication and/or information could be found. Many studies simply provided findings that the public health is at risk, and, in some cases, that communication efforts need to improve, but there has been no suggestion as to how this can actually be accomplished. Further research on the health impacts of air pollutants should therefore pay more attention to the aspect of science and risk communication in order to reach and inform the affected populations about the risks and enable the development and implementation of suitable preventive and protective interventions.

Although *health impacts* and *environmental protection, climate, sustainability* were the topics with the largest shares of the total and have been continuously represented with an increasing trend over time, they mostly only contained references to a necessary communication of the research results, but did not contain concrete approaches. Most of the work on communicating and informing about risks associated with air pollutants, as well as specific approaches on how communication in this context can be done, is represented by the topic of *risk communication*. Although this topic has also increased over time in publication numbers, especially since the 1990s, it still only represents the third-largest topic area in the sample overall and has decreased in topic proportion. This finding shows that there is still a lot of potential for further research on air pollutants in the subject of risk communication. Only through adequate communication and information of the results of air-pollutant research can the affected population be made aware of the existing risks and take measures to protect their health.

With a total amount of *n* = 84, a share of only 1% of our sample, references to UFP in the context of science communication about public health information are very scarce. By further manually analyzing the identified references to UFP, we only identified eight publications concerning communication or information towards UFP in our total sample [98–105] and only two of these references included specific approaches to examining appropriate communication methods that also included the affected population [98, 105]. This finding shows that, even though scientific data in UFP research already exists, such as the predictive models as recently researched by Saha et al. [112], there are still some research gaps in communicating scientific results on this fraction of PM, especially regarding the affected population and the non-scientific public.

This review was conducted to provide an overview of the published scientific literature on science communication of public-health information about risks associated with air pollutants, with specific reference to UFP. This should serve as a basis for analyzing whether, to what extent, and in which subject areas research has already been conducted on this topic. For further research, this could highlight existing approaches in the identified literature that can be used as a basis for developing or elaborating further research for science communication. Our results show that UFP have not been sufficiently researched in the topic area of PM with regard to communication and information; future research should shed more light on this topic. This also applies to the scientific communication of health and risk information.

### Limitations

First, our literature search results can only represent those articles that could be found based on our search terms. We revised and improved the search terms several times. We cannot guarantee, however, that we have found all relevant literature on the topics. Second, the databases used may also have systematic errors. As such, not all disciplines, journals, or studies are necessarily equally represented. Third, we included only original articles, reviews, and conference papers in English language in our search. “Gray literature” was not included. Consequently, it is possible that not all publications on the topics were mapped. Fourth, the inclusion of the term “public health”, which was identified as important in the preliminary test, resulted in many studies that had no link to science, health, or risk communication, or information as relates to air pollution. Fifth, due to the large number of publications, we manually analyzed only the top five titles, abstracts, and keywords for each identified topic. Although the top five articles per topic most accurately represented the topic, it cannot be ruled out that this did not capture all important publications on the topics or that further relevant approaches were not identified. Sixth, regarding the methodological procedure, although different topic-number solutions have been discussed, we could have integrated a more statistically based approach. For example, considering the robustness of topics across different models with varying topic numbers, as well as adding word- or topic-intrusion tests (e.g. Chang et al. [112]), could further validate the results of the model.

## Conclusions

This work aimed to generate an initial overview of the existing scientific literature on the topic of science communication of public-health risks related to air pollutants including UFP using STM. The results show that the existing literature can be categorized into five different topics: *Health impacts; environmental protection, climate, sustainability; risk communication; air quality management* and *prediction models.* The topic *risk communication,* with specific approaches for the communication of health risks from air pollution to the public, including the population in some of their analyses, seems especially underrepresented in the sample. Considering the fact that all other topics are still increasing in publication numbers, these results should be communicated more intensively and in a suitable form to the affected population to protect public health. In this context, a target-group-specific form of communication should be considered, and also the effectiveness of the communication efforts should be analyzed. It should also be emphasized that ultra-fine particles in particular have not yet been fully researched, and further scientific results are expected here as well, which should also be communicated adequately in the future. From a methodological point of view, the use of TPM has proven to be beneficial. Instead of a random sample, entire publications could be considered. This has not only provided valuable insights into the tradition and development of this particular field of research, but also offers the opportunity to explore the identified topics or topic groups in more detail. For example, a subsequent, in-depth examination focusing only on articles associated with a certain topic could illustrate the complexity of a given research focus. In addition, it would be interesting to analyze which combination of topics co-occur most frequently.

## Supplementary Information

**Additional file 1: Table 1.** Search strings. **Table 2.** Available metadata for Application Programming Interface retrieval. **Figure 1.** Different model diagnostics by number of topics. **Figure 2.** Flow diagram of study selection. **Table 3.** Topics identified through the STM with top 5 References per topic with reason if excluded.

**Additional file 2.**

## Data Availability

The data generated or analyzed during this study are included in this published article (and its supplementary information files). Additional file, Table 1 contains the exact search strings. Additional file, Table 2 includes Available metadata for API retrieval. Additional file, Fig. 1 shows the different model diagnostics by number of topics. Additional file, Fig. 2 shows the study selection process. Additional file, Table 3 includes all topics identified through the STM with top 5 References per topic. The complete datasets used and/or analyzed during the current study are available from the corresponding author on reasonable request.

## Abbreviations

PM: Particulate Matter
UFP: Ultra-fine Particles
WHO: World Health Organization
O_3_: Ozone
NO_2_: Nitrogen dioxide
SO_2_: Sulfur dioxide
CO: Carbon monoxide
Pb: Lead
EU: European Union
STM: Structured Topic Modelling
PRISMA-ScR: Preferred Reporting Items for Systematic Reviews and Meta-Analyses Extension for Scoping Reviews
API: Application Programming Interface
TPM: Topic Modelling
NLP: Natural Language Processing
LDA: Lancet Dirichlet Allocation
COPD: Chronic obstructive pulmonary disease
