# Supplementary material for: Science communication on the public health risks of air pollution: a computational scoping review from 1958 to 2022
Source: Arch Public Health. 2023 Feb 4;81:14. doi: 10.1186/s13690-023-01031-4 (PMC9898709; doi:10.1186/s13690-023-01031-4)
Supplement: Supplementary file 1 — Additional file 1: Table 1. Search strings. Table 2. Available metadata for Application Programming Interface retrieval. Figure 1. Different model diagnostics by number of topics. Figure 2. Flow diagram of study selection. Table 3. Topics identified through the STM with top 5 References per topic with reason if excluded. [file 13690_2023_1031_MOESM1_ESM.docx]

**Additional file**

**Table 1.** Search strings

| **Database** | **Searchterm** |
| --- | --- |
| PubMed | (“risk communication” [Title/Abstract]  OR “risk information” [Title/Abstract]  OR “public awareness” [Title/Abstract]  OR “public health” [Title/Abstract]  OR “public opinion” [Title/Abstract]  OR “health communication” [Title/Abstract]  OR “health information” [Title/Abstract]) AND  (“air pollution” [Title/Abstract]  OR “air pollutant” [Title/Abstract]  OR “air pollutants” [Title/Abstract]  OR “airborne pollutant” [Title/Abstract]  OR “airborne pollutants” [Title/Abstract]  OR “particulate matter” [Title/Abstract]  OR “air quality” [Title/Abstract]) |
| Scopus | TITLE-ABS((( “risk communication” OR “risk information” OR “public awareness” OR “public health” OR “public opinion” OR “health communication” OR “health information”) AND (“air pollution” OR “air pollutant” OR “air pollutants” OR “airborne pollutant” OR “airborne pollutants” OR “particulate matter” OR “air quality”))) AND (LIMIT-TO ( DOCTYPE,“ar” ) OR LIMIT-TO ( DOCTYPE,“re” ) OR LIMIT-TO ( DOCTYPE,“cp” )) AND (LIMIT-TO ( LANGUAGE,“English” ) OR LIMIT-TO ( LANGUAGE,“German” )) |

**Table 2.** Available metadata for Application Programming Interface retrieval

| Metadata | Description |
| --- | --- |
| doi | Digital Object Identifier |
| authors | First and last names of all authors |
| year | Year of publication |
| articletitle | Title of the publication |
| ptype | Type of publication |
| journal | Journal of publication |
| affiliations^1^ | Affiliation of all authors |
| countries^1^ | Country of all affiliations |
| volume | Volume of the journal |
| issue | Issue of the journal |
| pages | Pages of the publication |
| abstract | Abstract of the publication |
| keywords | Keywords of the publication |
| timescited^1^ | Number of citations of the publication |
| Pmid^2^ | PubMed ID of the publication |
| scopusID^1^ | Scopus ID of the publication |
| meshHeadings^2^ | MeSH Terms |
| chemNames^2^ | PubChem Identifiers |
| grantAgency^2^ | the institute acronym or mnemonic in the case of US PHS institutes or full organization name |
| grantNumber^2^ | the research grant or contract number (or both) that designates financial support by any agency of the United States Public Health Service, any institute of the National Institutes of Health, or any other organization |
| grantCountry^2^ | home country of the granting agency |
| nctID^2^ | NCT identifier |
| ^1^ only available for publications downloaded from Scopus  ^2^ only available for publications downloaded from PubMed | |


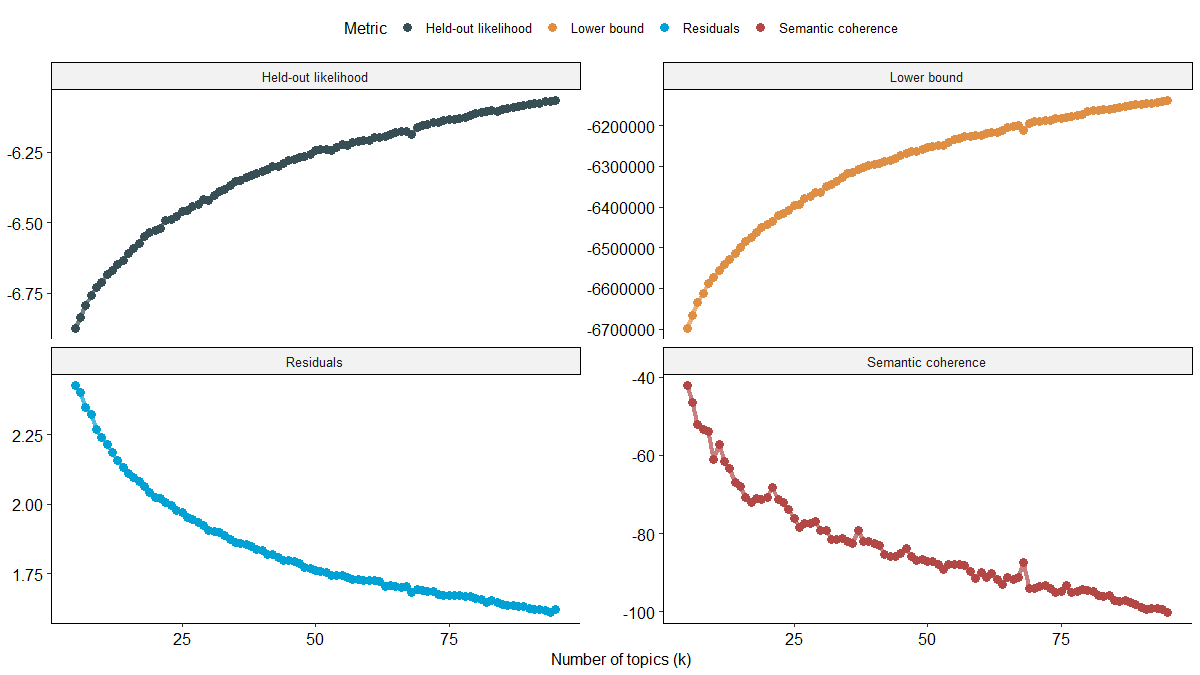


**Figure 1.** Different model diagnostics by number of topics

**Identification of studies via databases and registers**

Records removed *before screening*:

Duplicate records removed

(n = 182)

Records marked as ineligible by automation tools (n = 0)

Records removed for other reasons (n = 0)

Records identified from*:

Databases (n = 11052)

- Scopus: n = 7111
- PubMed: n = 3941

**Identification**

Records excluded**

(n = 4411)

Records screened

(n = 10870)

Abstracts sought for retrieval

(n = 6459)

Abstracts not retrieved

(n = 0)

**Screening**

Abstracts assessed for eligibility

(n = 6459)

Abstracts excluded:

non-English abstract (n = 1)

Abstract length (n = 35)

Abstracts included in review

(n = 6423)

**Included**

*Consider, if feasible to do so, reporting the number of records identified from each database or register searched (rather than the total number across all databases/registers).

**If automation tools were used, indicate how many records were excluded by a human and how many were

excluded by automation tools.

Details:

- Missing abstracts: n = 221
- Future publication: n = 1

Publication type: n = 41

*Modified adapted from:*  Page MJ, McKenzie JE, Bossuyt PM, Boutron I, Hoffmann TC, Mulrow CD, et al. The PRISMA 2020 statement: an updated guideline for reporting systematic reviews. BMJ 2021;372:n71. doi: 10.1136/bmj.n71

**Figure 2.** Flow diagram of study selection

**Table 3.** Topics identified through the STM with top 5 References per topic with reason if excluded

| **Topic** | **Author, Year** | **Title** | **Reason if excluded** |
| --- | --- | --- | --- |
| *Filters and Membranes* | Kim et al. 2019 | Effect of flow and humidity on indoor deposition of particulate matter | no communication or information |
|  | Kim et al. 2021 | Rational Process Design for Facile Fabrication of Dual Functional Hybrid Membrane of MOF and Electrospun Nanofiber towards High Removal Efficiency of PM2.5 and Toxic Gases | no communication or information |
|  | Zhou et al. 2020 | Polyacrylonitrile/polyimide composite sub-micro fibrous membranes for precise filtration of PM0.26 pollutants | no communication or information |
|  | Lv et al. 2019 | Ecofriendly Electrospun Membranes Loaded with Visible-Light-Responding Nanoparticles for Multifunctional Usages: Highly Efficient Air Filtration, Dye Scavenging, and Bactericidal Activity | no communication or information |
|  | Gui et al. 2022 | FeAl/Al2O3 porous composite microfiltration membrane for highly efficiency high‐temperature particulate matter capturing | no communication or information |
| *Preclinical analyses* | Zhang et al. 2021 | Shp2 regulates PM2.5-induced airway epithelial barrier dysfunction by modulating ERK1/2 signaling pathway | no communication or information |
|  | Zhu et al. 2019 | Inhibition of nuclear thioredoxin aggregation attenuates PM2.5-induced NF-κB activation and pro-inflammatory responses | no communication or information |
|  | Lin et al. 2022 | Exposure to PM2.5 induces neurotoxicity, mitochondrial dysfunction, oxidative stress and inflammation in human SH-SY5Y neuronal cells | no communication or information |
|  | Cao et al. 2021 | Tissue-protective effect of erdosteine on multiple-organ injuries induced by fine particulate matter | no communication or information |
|  | Wang et al. 2020 | Ambient particulate matter triggers dysfunction of subcellular structures and endothelial cell apoptosis through disruption of redox equilibrium and calcium homeostasis | no communication or information |
| *Risk communication* | Künzli and Perez 2009 | Evidence based public health - The example of air pollution | - |
|  | Huang and Yang 2019 | Beyond under the dome: an environmental documentary amplified public risk perception about air pollution in China | - |
|  | Liu and Zhang 2018 | Discursive constructions of scientific (Un)certainty about the health risks of China’s air pollution: A corpus-assisted discourse study | - |
|  | Fischer et al. 1973 | Elaboration of norms for assessment of the bacteriologic air pollution in public health institutions | - |
|  | Börner et al. 2015 | Exploring Mexican adolescents’ perceptions of environmental health risks: A photographic approach to risk analysis | - |
| *Pregnancy and birth* | Santri et al. 2021 | Associations of birth outcomes with air pollution and land use characteristics in the Greater Taipei Area | - |
|  | Requia et al. 2021 | Prenatal exposure to wildfire-related air pollution and birth defects in Brazil | - |
|  | Requia et al. 2022 | Increased preterm birth following maternal wildfire smoke exposure in Brazil | - |
|  | Boothe et al. 2014 | Residential traffic exposure and childhood leukemia: A systematic review and meta-analysis | - |
|  | Huang et al. 2018 | Investigation of association between environmental and socioeconomic factors and preterm birth in California | - |
| *Climate change and adaptions* | Poutiainen et al. 2013 | Civil society organizations and adaptation to the health effects of climate change in Canada | - |
|  | Nesbitt et al. 2017 | The social and economic value of cultural ecosystem services provided by urban forests in North America: A review and suggestions for future research | no communication or information |
|  | Wu and Le 2018 | Confronting the health-related challenges of climate change: Nursing education for the future | - |
|  | Su 2016 | An online tool for obesity intervention and public health | - |
|  | Mousavi et al. 2020 | Climate change and health in Iran: A narrative review 11 Medical and Health Sciences 1117 Public Health and Health Services | - |
| *Respiratory diseases* | Sakar et al. 2017 | Tuberculosis associated chronic obstructive pulmonary disease | - |
|  | Guo et al. 2020 | Prevalence and Risk Factors for COPD at High Altitude: A Large Cross-Sectional Survey of Subjects Living Between 2,100–4,700 m Above Sea Level | - |
|  | Rinne et al. 2006 | Relationship of pulmonary function among women and children to indoor air pollution from biomass use in rural Ecuador | - |
|  | Mishra et al. 2005 | Effects of cooking smoke and environmental tobacco smoke on acute respiratory infections in young Indian children | no communication or information |
|  | Agrawal 2012 | Effect of indoor air pollution from biomass and solid fuel combustion on prevalence of self-reported asthma among adult men and women in India: Findings from a nationwide large-scale cross-sectional survey | no communication or information |
| *Prediction models* | Kang et al. 2021 | Estimation of surface-level NO2 and O3 concentrations using TROPOMI data and machine learning over East Asia | no communication or information |
|  | Yafouz et al. 2022 | Comprehensive comparison of various machine learning algorithms for short-term ozone concentration prediction | - |
|  | Yan et al. 2017 | Two-Phase Neural Network Model for Pollution Concentrations Forecasting | - |
|  | Mishra and Goyal 2015 | Development of artificial intelligence based NO2 forecasting models at Taj Mahal, Agra | - |
|  | Wu and Lin 2019 | A novel optimal-hybrid model for daily air quality index prediction considering air pollutant factors | - |
| *Air quality management* | Viegas and Prista 2010 | Formaldehyde in indoor air: A public health problem? | - |
|  | Chan 1999 | Indoor Air Quality and the Law in Singapore | - |
|  | Longhurst et al. 2003 | Improving air quality through local air quality management. A critical review of British experience and practice | - |
|  | Evagelopoulos et al. 2021 | Smart air monitoring for indoor public spaces using mobile applications | - |
|  | Copabianco et al. 2013 | Impact of three interactive Texas state regulatory programs to decrease ambient air toxic levels | - |
| *Epidemiological analyses* | Phosri et al. 2018 | Effects of ambient air pollution on daily hospital admissions for respiratory and cardiovascular diseases in Bangkok, Thailand | - |
|  | Niu et al. 2016 | The association between ambient temperature and out-of-hospital cardiac arrest in Guangzhou, China | - |
|  | Kann et al. 2010 | Short-term association between sulfur dioxide and daily mortality: The Public Health and Air Pollution in Asia (PAPA) study | no communication or information |
|  | Kovats and Wilkinson 2004 | Contrasting patterns of mortality and hospital admissions during hot weather and heat waves in Greater London, UK | no communication or information |
|  | Cendon et al. 2006 | Air pollution effects on myocardial infarction | no communication or information |
| *Energy* | Deyette 2006 | An economic, employment, and environmental analysis of the Colorado renewable energy standard ballot initiative | no communication or information |
|  | Wiser et al. 2016 | The environmental and public health benefits of achieving high penetrations of solar energy in the United States | no communication or information |
|  | Zhang et al. 2019 | Installation planning in regional thermal power industry for emissions reduction based on an emissions inventory | no communication or information |
|  | Misila et al. 2020 | Thailand’s long-term GHG emission reduction in 2050: the achievement of renewable energy and energy efficiency beyond the NDC | - |
|  | Lu et al. 2019 | Carbon dioxide mitigation co-benefit analysis of energy-related measures in the Air Pollution Prevention and Control Action Plan in the Jing-Jin-Ji region of China | no communication or information |
| *Pollutant sources and concentrations* | Briggs and Long 2016 | Critical review of black carbon and elemental carbon source apportionment in Europe and the United States | no communication or information |
|  | Egami et al. 1990 | PM10 source apportionment study in Pleasant Valley, Nevada | no communication or information |
|  | Bernaudat and Nelson 2007 | Use of particle bound polycyclic aromatic hydrocarbon (PAHS) diagnostic ratios to assess pollution sources: Universal or local application? | no communication or information |
|  | Liu et al. 2020 | Variation trends and principal component analysis of nitrogen oxide emissions from motor vehicles in Wuhan City from 2012 to 2017 | no communication or information |
|  | Chow et al. 1990 | Applying the air quality source apportionments to geothermal power plant emissions | no communication or information |
