# Supplementary material for: Science communication on the public health risks of air pollution: a computational scoping review from 1958 to 2022
Source: Arch Public Health. 2023 Feb 4;81:14. doi: 10.1186/s13690-023-01031-4 (PMC9898709; doi:10.1186/s13690-023-01031-4)
Supplement: Supplementary file 2 — Additional file 2. [file 13690_2023_1031_MOESM2_ESM.docx]

**Statement to “Science communication on the public health risks of air pollution: a computational scoping review from 1958 to 2022”**

**What is known?**

Clean air is an essential factor for a healthy life but the air we breathe is often and at many places contaminated with various pollutants. This pollution composes one of the greatest environmental risks to human health. Every year, globally an estimated 4.2 million people die due to ambient air pollution. Air pollution refers not only to the ambient air, but also to that indoors, where many people spend most of the day. The effects of outdoor air pollution combined with indoor air pollution have been linked to even 6.7 million premature deaths per year [1]. For this reason, the World Health Organization has recently published new air quality guidelines which recommend maximum air quality levels. Based on these guidelines, many countries also derived statutory limits for air pollutants to protect human health [2].

Numerous studies have already examined the adverse health effects of air pollutants including for example respiratory or cardiovascular diseases [3-10]. In particular, vulnerable groups, for example pregnant women, elderly people, newborn and children, are often at risk from air pollutants [11]. In this context, it is not only important to achieve scientific knowledge, but also to accordingly communicate these results to the affected population.

**What does the study add?**

The scoping review helps to map the current state of knowledge on the communication and information of risks associated with air pollutants. The aim of this work is to find suitable approaches that can be expanded even further and also to adequately communicate the risks of ultrafine particles, which are currently the subject of further research, to the affected population. By using a computational approach with a Structured Topic Modelling the existing scientific literature is categorized and summarized so that an overview for identifying first approaches and research gaps for further and more detailed research is provided. Another benefit of this computer-assisted analysis is that it allows a significantly larger amount of data to be analyzed than with a manual approach.

**What the implications are for clinical practice, public health and/or research?**

Through the categorization and analysis of the existing scientific literature using Structured Topic Modeling, it was determined that communication and information about health risks from air pollutants identified in scientific research, including UFP, to the general public has received limited focus to date. Therefore, further studies are needed that also specifically include the affected population in their investigations. In addition, the effect or the comprehension of the developed tools or also of air quality indices should be examined. Another point to be considered is that the existing knowledge of the affected population is also decisive in this context. Depending on the level of knowledge and the target group, different measures for communication and information are required. The sources or media used for communication or from which information is obtained are also decisive and should be investigated in more detail in further scientific research. By identifying appropriate approaches to communicating risks associated with air pollutants, a contribution can be made towards the development of public awareness interventions and thus, in the long term, also to public health.

**References**

1. World Health Organization. *Air pollution*. Available online: <https://www.who.int/health-topics/air-pollution#tab=tab_1> (accessed on 10.01.2023

2. World Health Organization. *New WHO Global Air Quality Guidelines aim to save millions of lives from air pollution*. 2021; Available online: <https://www.who.int/news/item/22-09-2021-new-who-global-air-quality-guidelines-aim-to-save-millions-of-lives-from-air-pollution> (accessed 05.10.2021).

3. Munzel, T.; Gori, T.; Al-Kindi, S.; Deanfield, J.; Lelieveld, J.; Daiber, A.; Rajagopalan, S. *Effects of gaseous and solid constituents of air pollution on endothelial function.* Eur Heart J, 2018. **39**(38): p. 3543-50, doi: 10.1093/eurheartj/ehy481.

4. Mustafic, H.; Jabre, P.; Caussin, C.; Murad, M.H.; Escolano, S.; Tafflet, M.; Perier, M.C.; Marijon, E.; Vernerey, D.; Empana, J.P.; et al. *Main air pollutants and myocardial infarction: a systematic review and meta-analysis.* JAMA, 2012. **307**(7): p. 713-21, doi: 10.1001/jama.2012.126.

5. Munzel, T.; Sorensen, M.; Gori, T.; Schmidt, F.P.; Rao, X.; Brook, F.R.; Chen, L.C.; Brook, R.D.; Rajagopalan, S. *Environmental stressors and cardio-metabolic disease: part II-mechanistic insights.* Eur Heart J, 2017. **38**(8): p. 557-64, doi: 10.1093/eurheartj/ehw294.

6. Munzel, T.; Sorensen, M.; Gori, T.; Schmidt, F.P.; Rao, X.; Brook, J.; Chen, L.C.; Brook, R.D.; Rajagopalan, S. *Environmental stressors and cardio-metabolic disease: part I-epidemiologic evidence supporting a role for noise and air pollution and effects of mitigation strategies.* Eur Heart J, 2017. **38**(8): p. 550-6, doi: 10.1093/eurheartj/ehw269.

7. Cosselman, K.E.; Navas-Acien, A.; Kaufman, J.D. *Environmental factors in cardiovascular disease.* Nat Rev Cardiol, 2015. **12**(11): p. 627-42, doi: 10.1038/nrcardio.2015.152.

8. DeFranco, E.; Moravec, W.; Xu, F.; Hall, E.; Hossain, M.; Haynes, E.N.; Muglia, L.; Chen, A. *Exposure to airborne particulate matter during pregnancy is associated with preterm birth: a population-based cohort study.* Environ Health, 2016. **15**(6): p. 1-8, doi: 10.1186/s12940-016-0094-3.

9. van Rossem, L.; Rifas-Shiman, S.L.; Melly, S.J.; Kloog, I.; Luttmann-Gibson, H.; Zanobetti, A.; Coull, B.A.; Schwartz, J.D.; Mittleman, M.A.; Oken, E.; et al. *Prenatal air pollution exposure and newborn blood pressure.* Environ Health Perspect, 2015. **123**(4): p. 353-9, doi: 10.1289/ehp.1307419.

10. Ni, Y.; Szpiro, A.A.; Young, M.T.; Loftus, C.T.; Bush, N.R.; LeWinn, K.Z.; Sathyanarayana, S.; Enquobahrie, D.A.; Davis, R.L.; Kratz, M.; et al. *Associations of Pre- and Postnatal Air Pollution Exposures with Child Blood Pressure and Modification by Maternal Nutrition: A Prospective Study in the CANDLE Cohort.* Environ Health Perspect, 2021. **129**(4): p. 47004, doi: 10.1289/EHP7486.

11. United States Environmental Protection Agency. *Research on Health Effects from Air Pollution*. 2022; Available online: <https://www.epa.gov/air-research/research-health-effects-air-pollution#health-effects-vulnerable-pops> (accessed on 11.01. 2023).
